# Tobacco use and associated factors among Rwandan youth aged 15-34 years: Findings from a nationwide survey, 2013

**DOI:** 10.1371/journal.pone.0212601

**Published:** 2019-10-07

**Authors:** François Habiyaremye, Samuel Rwunganira, Clarisse Musanabaganwa, Marie Aimée Muhimpundu, Jared Omolo

**Affiliations:** 1 Department of Institute of HIV/AIDS Diseases Prevention and Control, Non-Communicable Diseases Division, Rwanda Biomedical Center, Kigali, Rwanda; 2 Rwanda Field Epidemiology and Laboratory Training Program, Kigali, Rwanda; Tribhuvan University Institute of Medicine, NEPAL

## Abstract

**Introduction:**

Use of tobacco and its products are the single most preventable cause of death in the world. The objective of this study was to determine the prevalence of current tobacco use and identify associated factors among Rwandans aged 15–34 years.

**Methods:**

This study involved secondary analysis of existing data from the nationally representative WHO STEPwise approach to Surveillance of non-communicable diseases (STEPS) conducted in 2013 to explore the prevalence of tobacco use and its associated factors in Rwanda. Data of 3,900 youth participants (15–34 years old) who had been selected using multistage cluster sampling during the survey was analyzed. The prevalence of current smoking along with socio-demographic characteristics of the sample were determined and multivariable logistic regression was employed to identify independent factors associated with current tobacco use.

**Results:**

The prevalence (weighted) of current tobacco use (all forms) was 8% (95%CI: 7.08–9.01). The prevalence was found to be significantly higher among males, young adults aged 24–34, youth with primary school education or less, those from Southern province, people with income (work in public, private organizations and self-employed) and young married adults. However, geographical location i.e. urban (7%) and rural (8%) settings did not affect prevalence of tobacco use. Factors that were found to be associated with current tobacco use through the multivariate analysis included being male, aged 25 years and above, having an income, and residing in Eastern, Kigali City and Southern Province compared to Western province.

**Conclusion:**

The association between smoking and socio-demographic characteristics among Rwandan youth identified in this study provides an opportunity for policy makers to tailor future tobacco control policies, and implement coordinated, high-impact interventions to prevent initiation of tobacco use among the youth.

## Introduction

Tobacco use is the single most preventable cause of death in the world[1]. The World Health Organization (WHO) estimates that there are nearly one billion smokers globally[2]. Every year, smoking accounts for more than 7 million preventable deaths worldwide[3]. The annual deaths are expected to reach 8 million by 2030 if, no cost effectiveness measures to reduce smoking are initiated [4]. Approximately, 80% of all the tobacco attributable deaths occur in low-middle income countries (LMICs)[5] such as Rwanda where tobacco use among adults is estimated to be 13%[6].

Without implementation and enforcement of effective tobacco control policies, smoking prevalence could increase to as high as 22% globally by 2030 [7]. The 2013 global burden of disease report estimates that deaths from tobacco use are among the top five causes of mortality in the East African Community (EAC) countries; Burundi, Kenya, Rwanda, South Sudan, Tanzania, and Uganda [8].

Current evidence shows that the path towards smoking and smoking addiction starts at a young age and strongly influences future adult smoking behaviour [9–10]. Although the Rwanda STEPS identified lifestyle risk factors associated with NCDs in the general adult population, no studies have been conducted to explore the link between tobacco use and socio-demographic factors among the youth in the country. This study was conducted to determine the prevalence of current tobacco use and identify associated factors among Rwandans aged 15–34 years. The Rwandan government policies cap the age of youth as persons aged between 14 to 35 years old[11].

This study was the first comprehensive analysis of the association of current tobacco use and selected socio-demographic characteristics for youth aged 15–34 in Rwanda, and provides evidence for a more targeted programmatic response to tobacco use among the youth in the country.

## Materials and methods

### Study design and study population

This was a cross sectional analytical study using secondary data collected from the nationally representative Non-Communicable Disease Risk Factors Survey, 2013 of Rwanda.

### Description of the Rwanda Non-Communicable Disease Risk Factors Survey

The STEPS survey was a population based cross-sectional study conducted in all 30 districts throughout the country from November 2012 to March 2013. The overall objective was to assess the magnitude of risk factors of selected Non-Communicable Diseases in the Rwandan population using the WHO STEPwise approach to surveillance (STEPS).

A multi-stage cluster sampling design was used to select a nationally representative sample. The WHO STEPwise approach was used to collect data using personal digital assistants (PDAs). These data included socio demographic and behavioural information; physical measurements such as height, weight, blood pressure and waist and hip circumference. Additionally, biochemical measurements were collected to assess total cholesterol, triglycerides levels, fasting blood glucose and urine albumin. In the initial survey, 7200 participants aged 15–64 years were enrolled.

### Data variables

Information on tobacco use was obtained by asking participants if they were current users of tobacco products. Current smokers were those who had smoked any tobacco product (such as cigarettes, cigars or rolled tobacco) in the previous 12 months. Additional information was collected on behavioral as well as physical and biochemical measurements.

### Statistical analyses

For this secondary data analysis, we extracted data from the STEPS survey for participants aged 15–34 years. Frequencies and percentages were used for descriptive analysis. The primary outcome i.e. “current tobacco use among participants aged 15 to 34 years” was modeled as a binary variable. We carried out weighted analysis to determine the prevalence of tobacco use and conducted bivariate analysis, assessing differences between categorical variables for significance using chi-square or Fisher’s exact test, as appropriate. Statistically significant variables were included in a multivariable model and analyzed using logistic regression to identify factors independently associated with current tobacco use. A p-value ≤ 0.05 was considered as significant. We used STATA (StataCorp 11.stata statistical software: Release 12. College Station, Tx:StataCorp LP.) for data analysis.

### Ethical considerations

The survey’s protocol was reviewed and approved by the Rwanda National Ethics Committee (RNEC) and the Centers for Disease Control and Prevention (CDC) Institutional Review Board. Consent was obtained from participants and no individually identifiable information was collected. The Rwanda Ministry of Health provided administrative approval to allow the secondary data analysis.

## Results

### Characteristics of participants

A total of 3900 participants aged 15–34 years were included in the analysis, of which 2405 (62%) were females. Overall 83% (3233) had primary education and below, 80% (944) lived in urban areas, 56% (2187) were married and 80% (3121) were engaged in some form of income generating employment. The socio-demographic characteristics of study participants by tobacco use are shown in Table 1.

**Table 1:** Socio-demographic characteristics by tobacco use (N = 3900).

Variables		Tobacco use		
Age group (in years)	N	% of tobacco users	% of non-tobacco users	p value
15–24	1511	4.4	95.5	0.001
25–34	2389	12.4	87.5	
**Gender**				
Men	1495	13.9	86	0.001
Women	2405	2.4	97.5	
**Level of Education**				
Primary school and below	3233	8.7	91.2	0.001
Secondary and High	669	4.7	95.2	
**Province**				
Eastern	972	8.8	91.1	0.001
Kigali	560	7.2	92.8	
Northern	651	5.4	94.5	
Southern	773	14	85.9	
Western	944	4.3	95.6	
**Employment status**				
No earnings	764	2.7	97.2	0.001
Earnings	3121	9.8	90	
**Residence**				
Rural	2956	8	92	0.89
Urban	944	7.8	92	
**Marital status**				
Married	2187	9.9	90	0.001
Others	1713	6.3	93.6	

### Tobacco use and associated factors

The prevalence (weighted) of current tobacco use (all forms) was 8% (95%CI: 7.08–9.01).

The prevalence of current tobacco use was significantly higher among males, young adults aged 24–34, youth whose highest education was primary school or below, those from Southern province (compared to Western), people with income and young married adults (Table 1).On the other hand, no statistically significant difference in prevalence was observed among study participants on the basis of residence.

The factors that were found to be associated with current tobacco use after multivariate analysis are shown in Table 2. Smoking was associated with being male, aged 25 years and above, residing in Eastern, Kigali City and Southern Province and having an income.

Education attainment was not associated with tobacco use (OR:1.2; 95%CI: [0.8–1.9]).

**Table 2:** Socio-demographic factors associated with tobacco use among Rwandans aged 15–34 years, 2012–2013.

Univariate analysis				Multivariate analysis		
Variables						
Age group (in yeas)	Crude OR	95% CI	P-value	Adjusted OR	95% CI	P value
15–24	1			1		
25–34	3	[2.2–4.2]	0.001	2.5	[1.7–3.6]	0.001
**Sex**						
Women	1			1		
Men	6.5	[5–8.6]	0.001	6.9	[5.2–9.1]	0.001
**Education**						
Secondary school and over	1			1		
Primary school and below	1.9	[1.26–2.8]	0.002	1.2	[0.8–1.9]	0.31
**Province**						
Western	1			1		
Eastern	2.1	[1.4–3.2]	0.001	2.3	[1.4–3.6]	0.001
Kigali	1.7	[1–2.9]	0.04	2.2	[1.2–3.8]	0.006
Northern	1.2	[0.7–2.1]	0.3NS	1.2	[0.7–2.1]	0.4NS
Southern	3.6	[2.4–5.3]	0.001	3.4	[2.2–5.1]	0.001
**Residence**						
Urban	1					
Rural	1.02	[0.7–1.3]	0.89			
**Employment status**						
No earning	1			1		
Earning	3.8	[2.4–6,1]	0.001	2.5	[1.4–4.2]	0.001
**Marital status**						
Others	1					
Married	0.6	[0.4–0.7]	0.001			

## Discussion

The WHO STEPS survey was conducted in Rwanda with the aim of providing national level estimates for various NCD risk factors (including tobacco use). To our knowledge, this is the first study conducted to assess tobacco use and associated factors among youth (15–34 years) in Rwanda. Our report using the Rwanda WHO STEPs database provides national-level estimates and information about the prevalence of tobacco use among youth and factors associated with its use in Rwanda. This secondary analysis targeted the youth which comprises over 60% of the Rwanda population and also represents the age at highest risk of tobacco initiation [10–12].

The findings of the study revealed that prevalence of current tobacco use was 2.4% among young women, 13.9% among young men while the overall prevalence (weighted) among the youth aged 15–35 was 8%. The results revealed quite similar differences in prevalence by gender among the youth and that of all adults observed during the Rwanda Demographic Health Survey RDHS 2014/2015 (2% women and 13% men)[13].

These findings are consistent with those of global estimates and other surveys which have found tobacco use to be more prevalent among men than women of all population groups[14]. The observation of higher tobacco consumption among males among youth has been consistently observed in many studies conducted in Rwanda and around the world. Examples include results from the Rwanda National health surveys[15][16], Rwanda NCDs risk factors surveys[6], the psychoactive substance abuse study[17], Ethiopian study on prevalence of tobacco use and associated factors[18], and the sociodemographic correlates of tobacco consumption in Rural Gujarat, India[19][20]. Furthermore, lower consumption of tobacco use among females, may be accentuated by a social desirability bias which attaches stigma to tobacco use among women. This study revealed that prevalence of tobacco use is lower among the younger youth aged 15–24 compared to older ones aged 25–34. It is likely that this lower prevalence may be associated with inadequate income and exposure to tobacco advertising. This further implies that there is a window of opportunity to intervene before the youth begin smoking.

The association between tobacco use and income in this study was consistent with findings of the World Health Survey on social determinants of smoking in low and middle-income countries which have shown that smoking is more prevalent among people with income compared to those who do not earn. This has been found to be significant after controlling for age, education and wealth in all settings except women of the low-income countries[21].

Tobacco taxation might also have a role which causes increase in cigarette market prices leading to lower affordability of the poorest wealth quintile. On the other hand, evidence also suggest that persons with higher incomes have the likelihood to avoid smoking initiation and use tobacco less[22]. This study shows that 8% of Rwandan youth are smokers compared to 13% of the general population (15–64 years)[13][16]. This relatively high prevalence among the youth raises concerns because of the negative long-term negative effects of tobacco use including death. The tobacco industry has continued to aggressively use cutting-edge edge technology to market their products and recruit more users among youth. Additionally, young people may have strong social networks which could influence initiation of tobacco consumption while making it difficult for those who have started to quit smoking. Therefore, behavioral interventions coupled with cessation programs would be an important step to support these young people to avoid or quit smoking[23].

This study revealed variations in tobacco use throughout Rwanda’s provinces. The highest prevalence was found in Eastern Province. This difference could be attributed to the availability of contraband cigarettes in this region and tobacco farming at a small scale for consumption purpose.

This study had a number of strengths. The secondary data analysis used data from the first nationwide study that allowed the assessing of factors associated with the current tobacco use in Rwandan youth aged 15–34 years. Additionally, the high overall response rate of 99.8% for Step 1, and 98.8% for Steps 2 and 3 in the primary study was very high and allowed the findings in the secondary analysis to be generalizable to all Rwandan youth aged 15–34 years.

The limitations of this study were that although it utilized data from the nationally representative Non-Communicable Disease Risk Factors Surveillance STEPS 2013 of Rwanda, a temporal relationship between the associated factors and tobacco use could not be established.

In addition, limited variables were collected during the primary data collection and it was not possible to assess other variables for this study.There is also the possibility of social desirability bias in reporting tobacco use, especially among women and might have led to underestimating of prevalence.

The study has some implications: Firstly, considering the health consequences of tobacco use, having 8% of Rwandan youth as tobacco users represents a substantial risk for morbidity and mortality unless preventive measures are instituted to mitigate the challenge. Cost effective interventions like health education should be prioritized to sensitize the youth on the risks associated with tobacco use. Sustained efforts through price controls and tax measures, comprehensive ban of tobacco smoking in public places, implementation and enforcement of bans to selling tobacco to and by minors in schools and families.

Secondly, the higher tobacco use among Rwandan youth implies that tobacco initiation occurs at a young age group. Implementing targeted interventions in education institutions (primary and secondary schools, all high learning institutions) should be initiated and strengthened early.

## Conclusions

This study shows that prevalence of tobacco smoking is high among Rwandan youth and is estimated to be at 8%. The study found that the factors significantly associated with tobacco use among the study population include age, gender, province of residence and employment status.

These findings provide an opportunity for policy makers, decision makers and relevant stakeholders to develop targeted interventions for young people while planning tobacco control interventions in general and implementing tobacco control policies in particular. Since, Rwandan youth are at risk of tobacco initiation and continuation, identifying ways and means of reaching them will be critical to the success or failure of the tobacco control program.
